# A Quality Improvement Study on Colonoscopy Wait Times in Underinsured Patients Following the COVID-19 Pandemic

**DOI:** 10.14309/ctg.0000000000000730

**Published:** 2024-06-25

**Authors:** Hong Gi Shim, Anuj Gupta, Andrew Fu, Ricardo Flores, Robert Simmons, Jonathan Steinberg, Arcelia Guerson-Gil, Yunhan Liao, Jie Yang, Joseph F. LaComb, Lionel S. D'Souza, Farah Monzur, Ellen Li, Alexandra Guillaume

**Affiliations:** 1 Division of Gastroenterology and Hepatology, Department of Medicine, Renaissance School of Medicine at Stony Brook University, Stony Brook, New York, USA;; 2 Department of Family, Population and Preventive Medicine, Renaissance School of Medicine at Stony Brook University, Stony Brook, New York, USA.

**Keywords:** quality improvement, health equity, COVID-19 pandemic, colonoscopy

## Abstract

**INTRODUCTION:**

The coronavirus disease 2019 (COVID-19) pandemic limited access to colonoscopy. To advance colorectal cancer health equity, we conducted a quality improvement study on colonoscopy wait times in 2019–2023 for underinsured (Medicaid, uninsured) compared with insured patients at an academic medical center providing colonoscopy for surrounding Federally Qualified Health Centers.

**METHODS:**

Retrospective chart reviews were performed on adult outpatient colonoscopies in the preintervention period (2019–2021). In 2022, an institutional grant funded bilingual patient navigation to reduce colonoscopy wait times. Postintervention data were collected prospectively from May 2022 to May 2023 in 2 phases. Multivariable regression analyses were conducted for colonoscopy wait times as a primary outcome.

**RESULTS:**

Analysis of 3,403 screening/surveillance and 1,896 diagnostic colonoscopies revealed significantly longer colonoscopy wait times for underinsured compared with insured patients after 2019. For screening/surveillance colonoscopies, wait time differences between underinsured and insured patients in the second postintervention phase were reduced by 34.21 days (95% confidence interval [CI]: 11.07–57.35) compared with the postpandemic period and by 56.36 days (95% CI: 34.16–78.55) compared with the first postintervention phase. For diagnostic colonoscopies, wait time differences in the second postintervention phase were reduced by 27.57 days (95% CI: 9.96–45.19) compared with the postpandemic period and by 20.40 days (95% CI: 1.02–39.77) compared with the first postintervention phase.

**DISCUSSION:**

Colonoscopy wait times were significantly longer for underinsured compared with insured patients following the COVID-19 pandemic. This disparity was partially ameliorated by patient navigation. Monitoring outpatient colonoscopy wait times in underinsured patients is important for advancing health equity.

## INTRODUCTION

Colonoscopy is regarded as the gold standard tool for screening, prevention, and diagnosis of colorectal cancer (CRC) (1). Adherence to recommended screening guidelines and prompt diagnostic evaluation of worrisome clinical findings are crucial to reduce the burden of CRC (1,2). However, significant disparities exist in rates, methods, and timeliness of CRC screening between underinsured and insured populations in the United States (3–5). Patients from ethnic minority groups or lower socioeconomic status are more likely to be underinsured and encounter financial and logistical barriers to completing colonoscopy (6,7). For underinsured patients, hospitals striving to provide equitable care are often the only gateway to colonoscopy, but long wait times are common due to insufficient resources to match the large referral base, which has recently grown with the lower age to start CRC screening in latest guidelines (8,9).

The coronavirus disease 2019 (COVID-19) pandemic presented unforeseen challenges to healthcare systems and created additional barriers to the timely completion of colonoscopies. Resource prioritization and procedural unit closures led to the postponement of colonoscopies during the pandemic. The backlog of postponed procedures put additional strains on the preexisting long wait times and raised concerns that such delays would disproportionately affect underinsured patients without alternative access. Current literature suggests that longer colonoscopy wait times may have significant clinical implications, such as diagnosing more advanced-stage CRCs with delayed colonoscopies after a positive fecal immunochemical test (FIT) (10,11).

We hypothesized that the COVID-19 pandemic created disproportionately longer colonoscopy wait times for underinsured patients and that a dedicated patient navigation (PN) would help reduce this disparity. PN has been described in the literature as an evidence-based, cost-effective intervention that improves adherence to CRC screening (12–14), increases the volume and timeliness of colonoscopy (13,15), and reduces disparities in healthcare accessibility (15–19). In this study, our aim was to understand patient characteristics and colonoscopy wait times of underinsured patients, implement a tailored PN program to reduce wait times, propose specific quality metrics with attainable goals, and create a system to assess our interventions and ensure sustained efforts on advancing health equity.

## METHODS

### Study design

A quality improvement study was designed based on the Plan-Do-Study-Act framework to reduce the disparity in colonoscopy wait times between underinsured and insured patients at Stony Brook University Hospital, serving a population of 1.5 million as a referral center for surrounding Federally Qualified Health Centers. This study was determined to be quality assured by Stony Brook Medicine (SBM) Division of Medical and Regulatory Affairs and exempt from informed consent by SBM Institutional Review Board (# IRB2022-00572). Handling of all data for this study was performed in accordance with quality assurance activity guidelines and regulations set by the SBM Division of Medical and Regulatory Affairs.

The study phases consisted of (i) a retrospective review of colonoscopy wait times in the preintervention period (2019–2021); (ii) a root cause analysis of colonoscopy wait times; (iii) implementation of a tailored, bilingual (English/Spanish) PN program for underinsured patients; and (iv) analysis of colonoscopy wait times in the postintervention period (May 2022–May 2023). Colonoscopy wait time was defined as the number of days between the date the colonoscopy appointment was scheduled in the system and the date the colonoscopy was completed. To calculate this, we first identified colonoscopies completed during specific time intervals and then recorded the dates of when these colonoscopies had been scheduled in our system. This metric reflects our endoscopy capacity and availability, with the assumption that appointments are scheduled at the earliest opportunity without significant delays by patients or providers, especially for diagnostic procedures. In essence, our calculation of wait times was intended to answer the question, “How long did each patient wait to complete a colonoscopy after the clinical decision was made to schedule it?”

Underinsured patients included those with Medicaid, Emergency Medicaid, or no insurance. The specific goal of the PN intervention was to reduce the disparity in colonoscopy wait times between the underinsured and insured patients from its peak level in the post-pandemic period down to at least similar to or lower than the baseline level in the prepandemic period. Based on the Specific-Measurable-Applicable-Realistic-Timely aim framework, we adopted a binary quality metric on colonoscopy wait times to monitor our progress. Given the lack of guidelines on acceptable colonoscopy wait times in the United States, we referred to the Canadian Association of Gastroenterology expert consensus statement on suggested wait times for colonoscopy based on the acuity category (20). Colonoscopy completion was considered “in-time” if the wait time was less than 180 days for screening or surveillance indications and less than 60 days for all other diagnostic indications. Because this metric was set based on expert consensus and could not be met in Canada even before the pandemic (21), we also measured colonoscopy wait times as a continuous variable in number of days. In addition, bowel preparation quality was measured, since inadequate bowel preparation could lead to colonoscopy cancellations, rescheduling, and prolonged wait times.

Based on this study design, data were divided into 5 time intervals: prepandemic (2019), pandemic (2020), postpandemic (2021), PN phase 1 (May 2022–October 2022), and PN phase 2 (November 2022–May 2023). Preintervention data on patient demographics, colonoscopy indication and wait times, and bowel preparation quality were collected to assess the change in colonoscopy wait times before, during, and after the pandemic. These data included all underinsured patients but 10% of randomly sampled insured patients due to some demographic and bowel preparation quality data requiring manual review not feasible for the large insured population during this period. Postintervention data included all underinsured and insured patients during this period. Although the PN intervention was implemented in May 2022, colonoscopy wait times measured in the first few months of the postintervention period likely reflected the clinical environment in which these colonoscopies had been scheduled before the intervention. Owing to this anticipated lag time to see the effect of the PN intervention, postintervention data were divided into the first 6 months (PN phase 1) and the last 7 months (PN phase 2).

### Statistical analysis

The primary outcomes of screening/surveillance and diagnostic colonoscopy wait times were measured separately as either continuous variables (days) or a binary quality metric (in-time or delayed). The secondary outcome of bowel preparation quality was recorded as a binary outcome (adequate or inadequate). Analyses of colonoscopy wait times were conducted separately for screening/surveillance and diagnostic colonoscopies. For univariate analyses of colonoscopy wait times as a continuous variable, the Pearson correlation coefficient was used to examine the linear correlation between wait times (days) and other continuous variables such as age. Wilcoxon rank-sum test (for variables with 2 categories) or Kruskal-Wallis test (for variables with more than 2 categories) was used to examine the marginal difference in wait times among categorical variables. For univariate analyses of colonoscopy wait times as categorical quality metrics (in-time or delayed), a χ^2^ test with exact *P* values based on Monte-Carlo simulation was used to examine the marginal association between wait times and categorical variables. Wilcoxon rank-sum test was used to examine the marginal difference in continuous variables (such as age in years) between in-time and delayed colonoscopies. Further multivariable regression models for each outcome (multiple linear regression models for wait time as a continuous variable and multivariable logistic regression for binary outcomes) were built to estimate the difference in wait times among insurance status (disparity) within each time period, as well as the difference in this disparity across time periods after adjusting for age (as a categorical variable: <45, 45–65 and >65 to model the possible nonlinear relationship between age and outcome), sex, race, ethnicity, and preferred language. CRC diagnosis as a proxy for symptoms was further adjusted in the multivariable regression model for diagnostic colonoscopies. The multivariable regression model for wait time categorized as ≥180 vs <180 days among screening/surveillance colonoscopy patients did not control for any other covariates due to the limited number of events of this binary outcome (22). Firth correction was applied to mitigate the bias caused by rare occurrences of underinsured screening/surveillance colonoscopies with delayed wait times in 2019 and 2020 (23). Statistical analysis was performed using SAS 9.4 (SAS Institute, Cary, NC). The significance level was set at 0.05.

## RESULTS

### Patient data and PN intervention

Data were collected on a total of 1,249 colonoscopies performed during the preintervention period (Table 1). This included all colonoscopies performed on the underinsured (n = 209) and 10% of the colonoscopies performed on the insured (n = 1,040). Data were collected on a total of 4,050 colonoscopies performed during the postintervention period (Table 1). This included all colonoscopies performed on the underinsured (n = 132) and all colonoscopies performed on the insured (n = 3,918).

**Table 1.** Patient demographics and colonoscopy characteristics of the study population, 2019–2023

	Prepandemic (2019) (n = 463)				Pandemic (2020) (n = 325)				Postpandemic (2021) (n = 461)				PN phase 1 (May 2022–October 2022) (n = 1,722)				PN phase 2 (November 2022–May 2023) (n = 2,328)				Preintervention (2019–2021) (n = 1,249)				Postintervention (May 2022–May 2023) (n = 4,050)			
	Underinsured	%	Insured (10% sample)	%	Underinsured	%	Insured (10% sample)	%	Underinsured	%	Insured (10% sample)	%	Underinsured	%	Insured	%	Underinsured	%	Insured	%	Underinsured	%	Insured (10% sample)	%	Underinsured	%	Insured	%
Sample size (n)	64	—	399	—	57	—	268	—	88	—	373	—	62	—	1,660	—	70	—	2,258	—	209	—	1,040	—	132	—	3,918	—
Age (y, median) (IQR)	54 (46–61)	—	59 (51–67)	—	54 (44–65)	—	59 (50–67)	—	52 (44–59)	—	57 (51–65)	—	53 (43–64)	—	58 (50–67)	—	52 (45–62)	—	58 (50–67)	—	52 (45–61)	—	58 (51–66)	—	52 (44–63)	—	58 (50–67)	—
Sex																												
Female	42	65.6	203	50.9	23	40.4	140	52.2	57	64.8	192	51.5	34	54.8	887	53.4	31	44.3	1,168	51.7	122	58.4	535	51.4	65	49.2	2,055	52.5
Male	22	34.4	196	49.1	34	59.6	128	47.8	31	35.2	181	48.5	28	45.2	773	46.6	39	55.7	1,090	48.3	87	41.6	505	48.6	67	50.8	1,863	47.5
Race																												
White																												
Not Hispanic/Latino	12	18.8	305	76.4	13	22.8	194	72.4	6	6.8	259	69.4	5	8.1	1,232	74.2	14	20.0	1,678	74.3	31	14.8	758	72.9	19	14.4	2,910	74.3
Hispanic/Latino	8	12.5	15	3.8	11	19.3	5	1.9	12	13.6	17	4.6	5	8.1	80	4.8	5	7.1	95	4.2	31	14.8	37	3.6	10	7.6	175	4.5
Missing ethnicity	0	0.0	9	2.3	1	1.8	3	1.1	0	0.0	6	1.6	0	0.0	18	1.1	0	0.0	36	1.6	1	0.5	18	1.7	0	0.0	54	1.4
Non-White																												
Not Hispanic/Latino	12	18.8	44	11.0	12	21.1	41	15.3	17	19.3	56	15.0	9	14.5	207	12.5	12	17.1	252	11.2	41	19.6	141	13.6	21	15.9	459	11.7
Hispanic/Latino	32	50.0	22	5.5	20	35.1	20	7.5	53	60.2	28	7.5	41	66.1	108	6.5	39	55.7	151	6.7	105	50.2	70	6.7	80	60.6	259	6.6
Missing ethnicity	0	0.0	1	0.3	0	0.0	0	0.0	0	0.0	1	0.3	1	1.6	6	0.4	0	0.0	11	0.5	0	0.0	2	0.2	1	0.8	17	0.4
Missing race	0	0.0	3	0.8	0	0.0	5	1.9	0	0.0	6	1.6	1	1.6	9	0.5	0	0.0	35	1.6	0	0.0	14	1.3	1	0.8	44	1.1
Ethnicity																												
Not Hispanic/Latino	24	37.5	350	87.7	25	43.9	235	87.7	23	26.1	315	84.5	14	22.6	1,440	86.7	26	37.1	1935	85.7	72	34.4	900	86.5	40	30.3	3,375	86.1
Hispanic/Latino	40	62.5	37	9.3	31	54.4	26	9.7	65	73.9	45	12.1	46	74.2	188	11.3	44	62.9	247	10.9	136	65.1	108	10.4	90	68.2	435	11.1
Missing	0	0.0	12	3.0	1	1.8	7	2.6	0	0.0	13	3.5	2	3.2	32	1.9	0	0.0	76	3.4	1	0.5	32	3.1	2	1.5	108	2.8
Preferred language																												
English	35	54.7	376	94.2	23	40.4	247	92.2	33	37.5	355	95.2	22	35.5	1,585	95.5	31	44.3	2,166	95.9	91	43.5	978	94.0	53	40.2	3,751	95.7
Non-English	29	45.3	23	5.8	34	59.6	21	7.8	55	62.5	18	4.8	40	64.5	72	4.3	39	55.7	91	4.0	118	56.5	62	6.0	79	59.8	163	4.2
Missing	0	0.0	0	0.0	0	0.0	0	0.0	0	0.0	0	0.0	0	0.0	3	0.2	0	0.0	1	0.0	0	0.0	0	0.0	0	0.0	4	0.1
Procedure indication																												
Diagnostic	48	75.0	193	48.4	39	68.4	136	50.7	59	67.0	167	44.8	30	48.4	570	34.3	32	45.7	622	27.5	146	69.9	496	47.7	62	47.0	1,192	30.4
Screening/surveillance	16	25.0	206	51.6	18	31.6	132	49.3	29	33.0	206	55.2	32	51.6	1,090	65.7	38	54.3	1,636	72.5	63	30.1	544	52.3	70	53.0	2,726	69.6
Wait time (d, median) (IQR)																												
All indications	63 (37–84)	—	50 (28–77)	—	56 (35–106)	—	31 (17.3–56)	—	116 (58–147)	—	55 (28.5–87)	—	125 (42–161)	—	51 (24–85)	—	77 (42–91)	—	55 (27–111)	—	70 (42–119)	—	48 (24–77)	—	85 (42–139)	—	53 (26–99)	—
Diagnostic	63 (41.3–84)	—	49 (22.5–75)	—	55 (35–105)	—	27 (14–49.8)	—	98 (62–154)	—	41 (19–73)	—	65.5 (34.5–155.3)	—	37 (18–68)	—	73.5 (42–91)	—	39 (17.8–63)	—	69.5 (42–113.8)	—	39 (18–68)	—	72.5 (35–112.3)	—	38 (18–66)	—
Screening/surveillance	70 (26.5–85.5)	—	53.5 (34.8–79.3)	—	62.5 (34–113.8)	—	41 (22–63.3)	—	126 (49.5–140)	—	65 (35.8–95.5)	—	149.5 (57–175)	—	61 (28–95)	—	77.5 (42–91)	—	70 (32.3–118)	—	79 (35–126)	—	54 (29–83)	—	90.5 (48–154)	—	65 (30–109)	—
Colonoscopy wait times																												
Diagnosis in time (<60 d)	23	47.9	126	65.3	21	53.8	115	84.6	14	23.7	114	68.3	15	50.0	405	71.1	14	43.8	456	73.3	58	39.7	355	71.6	29	46.8	861	72.2
Diagnosis delayed (≥60 d)	25	52.1	67	34.7	18	46.2	21	15.4	45	76.3	53	31.7	15	50.0	165	28.9	18	56.3	166	26.7	88	60.3	141	28.4	33	53.2	331	27.8
Screening/surveillance in time (<180 d)	16	100	203	98.5	18	100	131	99.2	28	96.6	202	98.1	27	84.4	1,078	98.9	37	97.4	1,610	98.4	62	98.4	536	98.5	64	91.4	2,688	98.6
Screening/surveillance delayed (≥180 d)	0	0	3	1.5	0	0	1	0.8	1	3.4	4	1.9	5	15.6	12	1.1	1	2.6	26	1.6	1	1.6	8	1.5	6	8.6	38	1.4
Bowel preparation quality																												
All indications–adequate	48	78.7	352	89.3	39	78.0	244	91.4	65	79.3	344	93.0	56	90.3	1,489	90.4	56	81.2	1985	89.6	152	78.8	940	91.2	112	85.5	3,474	90.0
All indications–inadequate	13	21.3	42	10.7	11	22.0	23	8.6	17	20.7	26	7.0	6	9.7	158	9.6	13	18.8	230	10.4	41	21.2	91	8.8	19	14.5	388	10.0
Missing	3	4.7	5	1.3	7	12.3	1	0.4	6	6.8	3	0.8	0	0	13	0.8	1	1.4	43	1.9	16	7.7	9	0.9	1	0.8	56	1.4
Diagnostic–adequate	36	80.0	164	85.9	24	70.6	121	89.6	47	85.5	148	89.2	27	90.0	504	89.0	28	87.5	513	84.9	107	79.9	433	88.0	55	88.7	1,017	86.9
Diagnostic–inadequate	9	20.0	27	14.1	10	29.4	14	10.4	8	14.5	18	10.8	3	10.0	62	11.0	4	12.5	91	15.1	27	20.1	59	12.0	7	11.3	153	13.1
Screening/surveillance–adequate	12	75.0	188	92.6	15	93.8	123	93.2	18	66.7	196	96.1	29	90.6	985	91.1	28	75.7	1,472	91.4	45	76.3	507	94.1	57	82.6	2,457	91.3
Screening/surveillance–inadequate	4	25.0	15	7.4	1	6.3	9	6.8	9	33.3	8	3.9	3	9.4	96	8.9	9	24.3	139	8.6	14	23.7	32	5.9	12	17.4	235	8.7
CRC incidence																												
New CRC found	2	3.1	4	1.0	2	3.5	3	1.1	0	0	1	0.3	0	0	9	0.5	0	0	16	0.7	4	1.9	8	0.8	0	0	25	0.6
No CRC found	62	96.9	395	99.0	55	96.5	265	98.9	88	100	372	99.7	62	100	1,651	99.5	70	100	2,242	99.3	205	98.1	1,032	99.2	132	100	3,893	99.4

CRC, colorectal cancer; IQR, interquartile range.

Patient characteristics including race, ethnicity, and preferred language were analyzed over the study period (Table 1). In the postintervention period, the insured panel consisted of 81.0% of patients of White/European ancestry, 11.4% of Hispanic/Latino ethnicity, and 95.8% speaking English as the preferred language. By contrast, the underinsured panel comprised 22.1% of patients of White/European ancestry, 69.2% of Hispanic/Latino ethnicity, and 40.2% speaking English as the preferred language. Similar demographic compositions were observed in the underinsured group and the randomly sampled 10% of the insured group in the preintervention period.

**Figure 1.**
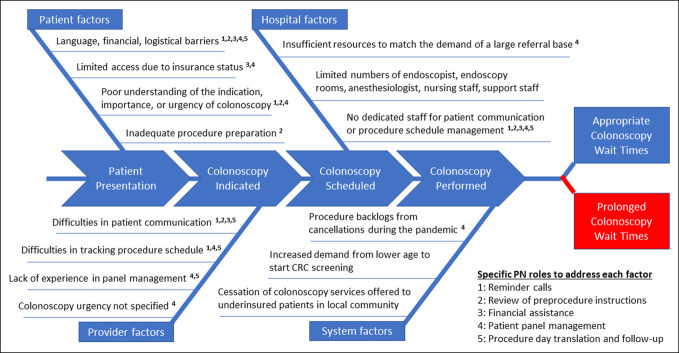
Fishbone diagram of factors contributing to colonoscopy wait times in underinsured patients.

A root cause analysis of factors affecting colonoscopy wait times was performed (Figure 1). To address modifiable factors, a tailored PN program for underinsured patients was implemented, and specific roles of the bilingual patient navigator were outlined (see Supplementary Appendix 1, http://links.lww.com/CTG/B151).

### Screening/surveillance colonoscopy wait times as a continuous variable

On univariate analyses (Table 2), age (as a categorical variable), insurance status, and colonoscopy time period were significantly associated with screening/surveillance colonoscopy wait times as a continuous variable (*P* < 0.05). Underinsured patients had a significantly longer median wait time than insured patients (median ± interquartile range [IQR]: 86.00 ± 96.00 days vs 62.00 ± 75.00 days, *P* < 0.0001). In addition, patient age (in years) was found to have a positive correlation with colonoscopy wait time (Pearson correlation coefficient = 0.0457, *P* = 0.0076).

**Table 2.** Univariate analysis of screening/surveillance and diagnostic colonoscopy continuous wait times (d)

Variable	Level	Screening/surveillance colonoscopy				Diagnostic colonoscopy			
		N	Median	IQR	*P* value^a^	N	Median	IQR	*P* value^a^
Age (as a categorical variable)	<45	126	67.00	81.00	**0.0018**	556	39.50	51.00	**0.0113**
	45–65	2,366	60.00	75.00		799	44.00	54.00	
	>65	911	69.00	76.00		541	39.00	53.00	
Sex	Female	1,691	62.00	76.00	0.2308	1,086	42.50	53.00	0.1281
	Male	1712	63.50	74.50		810	39.50	49.00	
Race	Not White	697	61.00	74.00	0.3482	499	48.00	53.00	**<0.0001**
	White	2,668	63.50	75.00		1,376	39.00	50.00	
Ethnicity	Hispanic or Latino	403	63.00	77.00	0.5135	366	50.50	60.00	**<0.0001**
	Not Hispanic or Latino	2,906	63.00	74.00		1,481	39.00	49.00	
Preferred language	English	3,196	63.00	74.00	0.4272	1,677	40.00	49.00	**<0.0001**
	Non-English	203	63.00	89.00		219	57.00	64.00	
Insurance	Insured	3,270	62.00	75.00	**<0.0001**	1,688	38.00	48.00	**<0.0001**
	Underinsured	133	86.00	96.00		208	70.00	70.50	
Colonoscopy period	Prepandemic	222	54.50	46.00	**<0.0001**	241	50.00	50.00	**<0.0001**
	Pandemic	150	43.50	43.00		175	31.00	40.00	
	Postpandemic	235	71.00	69.00		226	51.00	65.00	
	PN phase 1	1,122	62.00	69.00		600	38.00	50.50	
	PN phase 2	1,674	71.00	84.00		654	41.00	49.00	
CRC diagnosis	No	3,395	63.00	74.00	0.4950	1867	42.00	51.00	**0.0010**
	Yes	8	42.50	88.50		29	22.00	38.00	

CRC, colorectal cancer; IQR, interquartile range.

a *P* values were from Wilcoxon rank-sum test for variables having 2 categories and Kruskal-Wallis test for variables having more than 2 categories.

There were 38, 94, and 4 screening/surveillance colonoscopy patients with no race, ethnicity, and preferred language information. There were 21 and 49 diagnostic colonoscopy patients with no race and ethnicity information.

Significant P values are displayed in bold.

After adjusting for age (categorical), sex, race, ethnicity, and preferred language, the estimated differences in screening/surveillance colonoscopy wait times with 95% confidence interval (CI) between underinsured and insured patients during each colonoscopy period were significantly different in the postpandemic period (estimated difference = 36.54, 95% CI: 18.20–54.89) and PN phase 1 (estimated difference = 58.69, 95% CI: 41.52–75.86), but not in prepandemic or pandemic periods (Table 3). The estimated differences in wait times between underinsured and insured patients in PN phase 2 were reduced by 34.21 days compared with the post-pandemic period (95% CI: 11.07–57.35, *P* = 0.0038) and by 56.36 days compared with PN phase 1 (95% CI: 34.16–78.55, *P* < 0.0001) (Table 4).

**Table 3.** Estimated differences with 95% CI of continuous wait time (days) between underinsured and insured patients within each time period for screening/surveillance and diagnostic colonoscopies based on a multiple linear regression model after adjusting for covariate(s)

Level	Screening/surveillance colonoscopy^a^		Diagnostic colonoscopy^b^	
	Estimated differences between underinsured vs insured (95% CI)	*P* value^c^	Estimated differences between underinsured vs insured (95% CI)	*P* value^c^
Prepandemic	4.23 (−19.22 to 27.67)	**<0.0001**	9.53 (−3.00 to 22.06)	**<0.0001**
Pandemic	19.19 (−3.53 to 41.91)		**30.14 (15.80** to **44.47)**	
Postpandemic	**36.54 (18.20** to **54.89)**		**47.09 (35.08** to **59.10)**	
PN phase 1	**58.69 (41.52** to **75.86)**		**39.92 (25.19** to **54.65)**	
PN phase 2	2.33 (−12.74 to 17.40)		**19.52 (5.29** to **33.75)**	

CI, confidence interval; PN, patient navigation.

a Other covariates included in this model were age as a categorical variable, sex, race, ethnicity, and preferred language, and their corresponding estimated coefficients can be found in Supplementary Digital Content (see Appendix 5, http://links.lww.com/CTG/B151).

b Other covariates included in this model were age as a categorical variable, sex, race, ethnicity, preferred language, and CRC diagnosis, and their corresponding estimated coefficients can be found in Supplementary Digital Content (see Appendix 7, http://links.lww.com/CTG/B151).

c *P* value was from testing if the estimated difference is different across different time periods.

Significant P values are displayed in bold.

**Table 4.** Comparison of the estimated difference in screening/surveillance and diagnostic colonoscopy wait time (days) between underinsured and insured patients across time periods after adjusting for covariate(s)

Level	Screening/surveillance colonoscopy^a^		Diagnostic colonoscopy^b^	
	Estimated differences between underinsured vs insured (95% CI)	*P* value	Estimated differences between underinsured vs insured (95% CI)	*P* value
Prepandemic vs pandemic	−14.96 (−47.39 to 17.47)	0.3658	**−20.60 (−38.96** to **−2.24)**	**0.0279**
Prepandemic vs postpandemic	**−32.32 (−61.68** to **−2.96)**	**0.0310**	**−37.56 (−54.09** to **−21.03)**	**<0.0001**
Prepandemic vs PN phase 1	**−54.46 (-83.11** to **-25.81)**	**0.0002**	**−30.38 (−48.90** to **−11.87)**	**0.0013**
Prepandemic vs PN phase 2	1.90 (−25.65 to 29.45)	0.8927	−9.99 (−28.17 to 8.20)	0.2816
Pandemic vs postpandemic	−17.36 (−46.19 to 11.48)	0.2381	−16.96 (−34.76 to 0.85)	0.0620
Pandemic vs PN phase 1	**−39.50 (−67.61** to **−11.39)**	**0.0059**	−9.78 (−29.35 to 9.79)	0.3270
Pandemic vs PN phase 2	16.86 (−10.14 to 43.85)	0.2209	10.62 (−8.61 to 29.84)	0.2790
Postpandemic vs PN phase 1	−22.14 (−46.49 to 2.21)	0.0747	7.18 (−10.77 to 25.13)	0.4331
Postpandemic vs PN phase 2	**34.21 (11.07** to **57.35)**	**0.0038**	**27.57 (9.96** to **45.19)**	**0.0022**
PN phase 1 vs PN phase 2	**56.36 (34.16** to **78.55)**	**<0.0001**	**20.40 (1.02** to **39.77)**	**0.0391**

CI, confidence interval; PN, patient navigation.

a Other covariates included in the model were age as a categorical variable, sex, race, ethnicity, and preferred language. and their corresponding estimated coefficients can be found in Supplementary Digital Content (see Appendix 5, http://links.lww.com/CTG/B151).

b Other covariates included in this model were age as a categorical variable, sex, race, ethnicity, preferred language, and CRC diagnosis, and their corresponding estimated coefficients can be found in Supplementary Digital Content (see Appendix 7, http://links.lww.com/CTG/B151).

Significant P values are displayed in bold.

### Diagnostic colonoscopy wait times as a continuous variable

On univariate analyses (Table 2), age (as a categorical variable), race, ethnicity, preferred language, insurance status, colonoscopy time period, and CRC diagnosis on colonoscopy were significantly associated with diagnostic colonoscopy wait times (*P* < 0.05). Patient age (in years) was not significantly correlated with diagnostic colonoscopy wait times.

**Figure 2.**
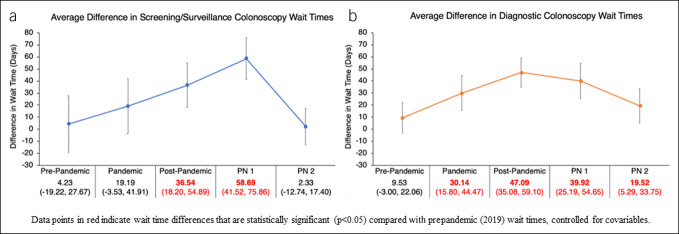
Estimated wait time differences between underinsured and insured patients for (**a**) screening/surveillance and (**b**) diagnostic colonoscopy.

On multiple linear regression analysis (see Supplementary Appendix 7, http://links.lww.com/CTG/B151), age (as a categorical variable) and CRC diagnosis were significant (*P* < 0.05), but race, ethnicity, and language preference did not reach significance. After controlling for age (as a categorical variable), race, sex, ethnicity, preferred language, and CRC diagnosis, the estimated differences in diagnostic colonoscopy wait times between underinsured and insured patients were significantly different in the pandemic, postpandemic, PN phase 1, and PN phase 2 periods, but not in the prepandemic period (Figure 2, Table 3). The estimated differences in wait times between underinsured and insured patients in PN phase 2 were significantly reduced by 27.57 days compared with the postpandemic period (95% CI: 9.96–45.19, *P* = 0.0022) and by 20.40 days compared with PN phase 1 (95% CI: 1.02–39.77, *P* = 0.0391) (Figure 2, Table 4).

### Colonoscopy wait times as a binary quality metric outcome (in-time vs delayed)

Univariate analysis (see Supplementary Appendix 2, http://links.lww.com/CTG/B151) revealed that only insurance status was significantly associated with screening/surveillance colonoscopy wait times as a binary outcome (*P* = 0.0035), with only 1.4% of insured and 5.3% of underinsured screening/surveillance colonoscopies being delayed for over 180 days. There was no evidence that such association differs across different time periods.

By contrast, univariate analysis of diagnostic colonoscopy wait times as a binary outcome (see Supplementary Appendix 2, http://links.lww.com/CTG/B151) revealed significant associations with sex, race, ethnicity, preferred language, colonoscopy time period, and CRC diagnosis on colonoscopy, in addition to insurance status (*P* < 0.05), with 28.0% of insured and 58.2% of underinsured diagnostic colonoscopies being delayed for over 60 days. Among 29 patients who were diagnosed with CRC on diagnostic colonoscopy, 26 (89.7%) had a wait time of less than 60 days. By contrast, 1,277 of 1,867 patients (68.4%) who were not diagnosed with CRC on diagnostic colonoscopy had a wait time of less than 60 days. The odds ratio (OR) of underinsured patients having delayed diagnostic colonoscopy (wait time ≥60 days) compared with insured patients within each time period (Table 5) peaked in the postpandemic period (OR = 5.94, 95% CI: 2.88–12.24), but the differences in ORs between time periods did not reach a statistical significance based on a multivariable logistic regression model after controlling for age (as a categorical variable), sex, race, ethnicity, preferred language, and CRC diagnosis on colonoscopy.

**Table 5.** OR of having a delayed screening/surveillance colonoscopy (wait time ≥180 d) and diagnostic colonoscopy (wait time ≥60 days) in the underinsured vs insured based on a multivariable logistic regression model (after adjusting for covariates)

Level	Screening/surveillance colonoscopy^a^		Diagnostic colonoscopy^b^	
	OR (95% CI) between underinsured vs insured	*P* value^c^	OR (95% CI) between underinsured vs insured	*P* value^c^
Prepandemic	1.76 (0.08–38.62)	0.1639	1.65 (0.84–3.24)	0.0796
Pandemic	2.37 (0.09–64.72)		**4.17 (1.81**–**9.61)**	
Postpandemic	2.37 (0.35–16.07)		**5.94 (2.88**–**12.24)**	
PN phase 1	**17.25 (5.83**–**51.02)**		2.21 (1.00–4.88)	
PN phase 2	2.43 (0.45–13.27)		**3.15 (1.46**–**6.80)**	

CI, confidence interval; PN, patient navigation; OR, odds ratios.

a Firth correction was applied to mitigate the bias caused by rare events in having colonoscopy wait time ≥180 days for underinsured patients. The number of underinsured patients having colonoscopy wait time ≥180 days in the prepandemic and pandemic periods was 0.

b Other covariates included in the model were age as a categorical variable, sex, race, ethnicity, preferred language, and CRC diagnosis, and complete results can be found in Supplementary Digital Content (see Appendix 8, http://links.lww.com/CTG/B151).

c *P* value was from testing if the difference in risk of having a delayed screening/surveillance or diagnostic colonoscopy between underinsured and insured patients was significantly different across different time periods.

### Bowel preparation quality for colonoscopy

For screening/surveillance colonoscopies, univariate analyses (see Supplementary Appendix 3, http://links.lww.com/CTG/B151) showed that age (in years) and insurance status were significantly associated with bowel preparation quality (*P* < 0.05), with 91.74% of insured and only 79.69% of underinsured patients having adequate bowel preparation. In a multivariable logistic regression model (Table 6), bowel preparation quality varied significantly over time periods. For diagnostic colonoscopies, univariate analysis showed that only sex was significantly associated with bowel preparation quality (*P* = 0.0027). Adequate bowel preparation was observed in 87.24% of insured and 82.65% of underinsured patients undergoing diagnostic colonoscopy, but this difference did not reach statistical significance. The differences in the OR of having inadequate bowel preparation between insured and underinsured patients across time periods did not reach a statistical significance.

**Table 6.** OR for inadequate bowel preparation between underinsured and insured with their corresponding 95% CIs for screening/surveillance and diagnostic colonoscopy across time periods based on a multivariable logistic regression model after adjusting for covariate(s)

Level	Screening/surveillance colonoscopy^a^		Diagnostic colonoscopy^b^	
	OR (95% CI) between underinsured vs insured	*P* value^c^	OR (95% CI) between underinsured vs insured	*P* value^c^
Prepandemic	**4.56 (1.28**–**16.24)**	**0.0380**	1.34 (0.55–3.24)	0.1876
Pandemic	0.95 (0.11–8.16)		**3.50 (1.28**–**9.59)**	
Postpandemic	**12.47 (4.08**–**38.12)**		1.25 (0.49–3.23)	
PN phase 1	1.15 (0.33–4.04)		0.79 (0.22–2.85)	
PN phase 2	**3.52 (1.56**–**7.93)**		0.67 (0.22–2.07)	

CI, confidence interval; PN, patient navigation; OR, odds ratios.

a Other covariates included in the model were age as a categorical variable, sex, race, ethnicity, and preferred language, and the complete model results can be found in Supplementary Digital Content (see Appendix 6, http://links.lww.com/CTG/B151).

b Other covariates included in the model were age as a categorical variable, sex, race, ethnicity, preferred language, and CRC diagnosis, and the complete model results can be found in Supplementary Digital Content (see Appendix 9, http://links.lww.com/CTG/B151).

c *P* value was from testing if the difference in the risk of having inadequate prep quality between underinsured and insured was significantly different across different time periods.

Significant P values are displayed in bold.

## DISCUSSION

Faculty and trainees in our gastroenterology division initiated this study because of prolonged colonoscopy wait times in underinsured patients perceived by our clinicians in practice following the COVID-19 pandemic. We chose thresholds for colonoscopy wait times as a binary outcome based on the Canadian Association of Gastroenterology consensus statement (20) due to the lack of guidelines on colonoscopy wait times in the United States. However, because Canadian institutions failed to meet these metrics even before the pandemic (21), we also measured colonoscopy wait times as continuous variables. The acceptable colonoscopy wait time for any select indication remains unclear. Data on colonoscopy following a positive FIT indicate that prolonged wait times are associated with more advanced stage of CRC at diagnosis (10,11). Moreover, a prediction model showed that delays in screening colonoscopy beyond 4–6 months following the pandemic would significantly increase advanced CRC cases and mortality if delays persist beyond 12 months (24).

In our study population, only 1.4% of insured and 5.3% of underinsured patients undergoing screening/surveillance colonoscopy exceeded the recommended wait time. However, 28.0% of insured and 58.2% of underinsured patients undergoing diagnostic colonoscopy exceeded the recommended wait time. Multivariable regression analyses of diagnostic colonoscopy wait times, measured as continuous or binary categorical variables, demonstrated a significant association with insurance status. However, multivariable regression analyses of screening/surveillance colonoscopy wait time demonstrated a significant association with insurance status, only when measured as a continuous variable. Interestingly, a recent study from a Veterans Administration hospital, where insurance status was constant among all patients, also reported increased wait times following the pandemic (25).

To examine whether the pandemic or bilingual PN intervention was associated with changes in wait times, estimated wait time differences between underinsured and insured patients were compared within and between time periods. Multivariable regression analyses indicated that while estimated wait time differences for screening/surveillance colonoscopy between underinsured and insured groups did not reach statistical significance in the prepandemic period, these differences were significant in the postpandemic period and PN phase 1. For diagnostic colonoscopy, estimated wait time differences between underinsured and insured groups did not reach statistical significance in the prepandemic period, but these differences were significant with longer wait times for underinsured patients in all subsequent periods. However, the disparity in diagnostic colonoscopy wait times was ameliorated in PN phase 2, as the estimated wait time differences in this period was no longer significantly different from the prepandemic period.

When diagnostic colonoscopy wait times were measured as a binary outcome, the estimated differences between underinsured and insured patients were not significant across time periods, indicating that it was difficult to perform a significant portion of underinsured diagnostic colonoscopies within 60 days with our current resources even with the PN intervention. A possible explanation is that at the start of the postintervention period, there was a backlog of underinsured patients with diagnostic colonoscopy wait times already far exceeding 60 days. Fortunately, 89.7% of patients diagnosed with CRC had wait times less than 60 days. We speculate this is because our gastroenterologists used clinical judgment to prioritize colonoscopy for patients with alarming symptoms suggestive of CRC (e.g., evidence of gastrointestinal blood loss).

Because the PN intervention was single-armed and not randomized, we cannot be certain that the PN intervention was causal in reducing wait times following the height of the pandemic. Reduction of wait time disparity may be secondary to the role of PN in addressing high no-show rates in underinsured patients and reducing the number of unused procedural slots, as there was a significant increase in the total number of underinsured colonoscopies performed in the postintervention period (n = 132), compared with the prepandemic (n = 64), pandemic (n = 57), and postpandemic (n = 88) periods. On the other hand, our analysis suggested PN had a limited role in improving bowel preparation quality in underinsured compared with insured patients, despite multiple reminder calls.

Limitations of this study included the study being performed at a single medical center and collecting data on only 10% of randomly selected insured patients in the preintervention period but on all insured patients in the postintervention period. To mitigate the effect of this sampling process, sensitivity analyses were conducted by repeating multivariable analyses with multiple 10% and 50% sampling of the data from the postintervention period, which showed little effect on estimated differences between 10%, 50%, and 100% sampling.

Based on results of this study, plans are being made to continue the PN intervention, which would incur a higher cost to the hospital once research funds are exhausted. There is also a clear need to increase overall procedural capacity for underinsured patients by recruiting additional endoscopists. In the meantime, reducing diagnostic colonoscopy wait times for the underinsured could result in prolonged wait times for screening/surveillance colonoscopy and decreased clinical revenue. A risk stratification strategy using noninvasive CRC screening tools in asymptomatic patients referred for screening colonoscopy may be useful to address this issue. This 2-tiered approach is currently used by the state-funded, free CRC screening program in the New York State that first offers FIT, followed by colonoscopy at no cost to the patient if the FIT is positive. In conclusion, we recommend that both screening/surveillance and diagnostic colonoscopy wait times are monitored as important quality metrics for advancing CRC health equity.

## CONFLICTS OF INTEREST

**Guarantor of the article:** Alexandra Guillaume, MD.

**Specific author contributions:** H.G.S.: study planning and design, collecting and interpreting the data, drafting and editing the manuscript; A.G. and A.F.: study planning and design, collecting and interpreting the data, editing the manuscript; R.F. Jr: patient navigator, collecting and interpreting the data, editing the manuscript; R.S., J.S., A.G.: collecting the data, editing the manuscript; Y.L. & J.Y.: statistical analysis of the data, interpreting the data, editing the manuscript; J.F.L., L.D., and F.M.: study planning and design, editing the manuscript; E.L.: study planning and design, collecting and interpreting the data, editing the manuscript; A.G.: study planning and design, interpreting the data, editing the manuscript.

**Financial support:** Clinical Research Award in the Stony Brook School of Medicine Targeted Research Opportunity Program. The funder had no role in carrying out the study or writing the manuscript.

**Potential competing interests:** None to report.

**IRB approval statement:** This study was approved by the Stony Brook University Institutional Review Board (IRB # IRB2022-00572).

**Study Highlights**

**WHAT IS KNOWN**

✓ The coronavirus disease 2019 pandemic disrupted colonoscopy services and caused longer colonoscopy wait times in the postpandemic period.
✓ Patient navigation improves colonoscopy access and health disparities in underinsured patients.
✓ Longer diagnostic colonoscopy wait times following a positive fecal immunochemical test are associated with a more advanced colorectal cancer stage at diagnosis.
✓ Colonoscopy wait times are not routinely measured as a quality metric in the United States.

**WHAT IS NEW HERE**

✓ Applying quality improvement approaches to monitoring and improving colonoscopy wait times in underinsured patients following the coronavirus disease 2019 pandemic advances health equity.

## Supplementary Material
