# Supplementary material for: A Quality Improvement Study on Colonoscopy Wait Times in Underinsured Patients Following the COVID-19 Pandemic
Source: Clin Transl Gastroenterol. 2024 Jun 25;15(9):e1. doi: 10.14309/ctg.0000000000000730 (PMC11421721; doi:10.14309/ctg.0000000000000730)
Supplement: Supplementary file 1 [file ct9-15-e1h-s001.docx]

**Appendix 1. Patient Navigation Protocol for Colonoscopy in Underinsured Patients.**

| **Patient Navigator Roles** |
| --- |
| 1. Perform reminder calls to underinsured patients 2 weeks, 1 week, and 2 days before the scheduled endoscopy procedure.    1. Confirm the appointment time and date.    2. Confirm the patient’s planned attendance to the procedure.    3. Provide contact information for any further questions. |
| 1. Review standard pre-procedure dietary, medication, and bowel preparation instructions.    1. Confirm the patient’s understanding of required preparation instructions.    2. Provide verbal, printed, or electronic instructions as necessary.    3. Refer to appropriate resources for assistance with prescriptions, escorts, or transportation.    4. Verify any specific needs for periprocedural medication management (e.g. anticoagulation or hypoglycemic agents) and refer to appropriate providers.    5. Relay any other clinical questions or concerns to the responsible provider. |
| 1. Review financial assistance program and refer to appropriate services as necessary. |
| 1. Facilitate underinsured endoscopy management.    1. Maintain an active waitlist of urgent procedures to be moved up to the next available opening.    2. Identify any rescheduling or cancellations early and notify the responsible provider.    3. Facilitate filling of open spots with patients in consultation with responsible providers.    4. Notify and confirm with the patients for any rescheduled procedures. |
| 1. Attend underinsured procedure sessions.    1. Provide translation on the day of the procedure as necessary.    2. Assist patients with scheduling appropriate recalls and follow-ups after the procedure.    3. Provide outreach to any patient who did not show for an appropriate follow-up. |

**Appendix 2. Univariate analysis of screening/surveillance and diagnostic colonoscopy binary metric outcomes (in time: <180 days, not in time: ≥180 days for screening/surveillance colonoscopy; in time: <60 days, not in time: ≥60 days for diagnostic colonoscopy).**

| **Variable** | **Level** | **Screening/surveillance colonoscopy** | | | | **Diagnostic colonoscopy** | | | |  |
| --- | --- | --- | --- | --- | --- | --- | --- | --- | --- | --- |
|  |  | **Total (N=3,403)** | **≥180 days (N=53)** | **<180 days (N=3,350)** | **P-value*** | **Total (N=1,896)** | **≥60 days**  **(N=593)** | **<60 days (N=1,303)** | **P-value*** |  |
| Age (in years) |  | 58.62 ± 14.50 | 57.00 ± 16.72 | 58.74 ± 14.39 | 0.4376 | 56.0 ± 24.83 | 56.0 ± 23.00 | 56.0 ± 25.00 | 0.8747 |  |
| Age (as a categorical variable) | <45 | 126 (3.70%) | 3 (2.38%) | 123 (97.62%) | 0.7295 | 556 (29.32%) | 164 (29.50%) | 392 (70.50%) | 0.3049 |  |
|  | 45-65 | 2366 (69.53%) | 35 (1.48%) | 2331 (98.52%) |  | 799 (42.14%) | 265 (33.17%) | 534 (66.83%) |  |  |
|  | >65 | 911 (26.77%) | 15 (1.65%) | 896 (98.35%) |  | 541 (28.53%) | 164 (30.31%) | 377 (69.69%) |  |  |
| Gender | Female | 1691 (49.69%) | 24 (1.42%) | 1667 (98.58%) | 0.5758 | 1086 (57.3%) | 360 (33.2%) | 726 (66.9%) | **0.0444** |  |
|  | Male | 1712 (50.31%) | 29 (1.69%) | 1683 (98.31%) |  | 810 (42.7%) | 233 (28.8%) | 577 (71.2%) |  |  |
| Race | Not White | 697 (20.71%) | 15 (2.15%) | 682 (97.85%) | 0.1622 | 499 (26.6%) | 194 (38.9%) | 305 (61.1%) | **<.0001** |  |
|  | White | 2668 (79.29%) | 37 (1.39%) | 2631 (98.61%) |  | 1376 (73.4%) | 394 (28.6%) | 982 (71.4%) |  |  |
| Ethnicity | Hispanic or Latino | 403 (12.18%) | 10 (2.48%) | 393 (97.52%) | 0.1260 | 366 (19.8%) | 160 (43.7%) | 206 (56.3%) | **<.0001** |  |
|  | Not Hispanic or Latino | 2906 (87.82%) | 41 (1.41%) | 2865 (98.59%) |  | 1481 (80.2%) | 419 (28.3%) | 1062 (71.7%) |  |  |
| Preferred Language | English | 3196 (94.03%) | 48 (1.50%) | 3148 (98.50%) | 0.3678 | 1677 (88.5%) | 486 (29.0%) | 1191 (71.0%) | **<.0001** |  |
|  | Non-English | 203 (5.97%) | 5 (2.46%) | 198 (97.54%) |  | 219 (11.6%) | 107 (48.9%) | 112 (51.1%) |  |  |
| Insurance | Insured | 3270 (96.09%) | 46 (1.41%) | 3224 (98.59%) | **0.0035** | 1688 (89.0%) | 472 (28.0%) | 1216 (72.0%) | **<.0001** |  |
|  | Underinsured | 133 (3.91%) | 7 (5.26%) | 126 (94.74%) |  | 208 (11.0%) | 121 (58.2%) | 87 (41.8%) |  |  |
| Colonoscopy Period | Pre-pandemic | 222 (6.52%) | 3 (1.35%) | 219 (98.65%) | 0.8519 | 241 (12.7%) | 92 (38.2%) | 149 (61.8%) | **<.0001** |  |
|  | Pandemic | 150 (4.41%) | 1 (0.67%) | 149 (99.33%) |  | 175 (9.2%) | 39 (22.3%) | 136 (77.7%) |  |  |
|  | Post-pandemic | 235 (6.91%) | 5 (2.13%) | 230 (97.87%) |  | 226 (11.9%) | 98 (43.4%) | 128 (56.6%) |  |  |
|  | PN phase 1 | 1122 (32.97%) | 17 (1.52%) | 1105 (98.48%) |  | 600 (31.7%) | 180 (30.0%) | 420 (70.0%) |  |  |
|  | PN phase 2 | 1674 (49.19%) | 27 (1.61%) | 1647 (98.39%) |  | 654 (34.5%) | 184 (28.1%) | 470 (71.9%) |  |  |
| CRC Diagnosis | No | 3395 (99.76%) | 53 (1.56%) | 3342 (98.44%) | 1.0000 | 1867 (98.5%) | 590 (31.6%) | 1277 (68.4%) | **0.0129** |  |
|  | Yes | 8 (0.24%) | 0 (0.00%) | 8 (100.00%) |  | 29 (1.5%) | 3 (10.3%) | 26 (89.7%) |  |  |
| * For continuous variables, p-values were based on Wilcoxon rank sum test; for categorical variables, p-values were based on Chi-squared test with exact p-value from Monte Carlo simulation.  Note: Median ± IQR were reported for continuous variables; row percentages were reported for categorical variables.  Note: 38, 94, and 4 screening/surveillance colonoscopy patients had no race, ethnicity, and preferred language information. 21 and 49 diagnostic colonoscopy patients had no race and ethnicity information. | | | | | | | | | | |

**Appendix 3: Descriptive table of screening/surveillance colonoscopy patients’** **characteristics and clinical variables by prep quality.**

| **Variable** | **Nmissing** | **Level** | **Total (N=3,359)** | **Adequate (N=3,066)** | **Inadequate (N=293)** | **P-value*** |
| --- | --- | --- | --- | --- | --- | --- |
| Age (in years) | 0 |  | 58.62 ± 14.50 | 58.00 ± 14.93 | 60.98 ± 14.00 | **0.0012** |
| Age (as a categorical variable) | 0 | <45 | 126 (3.70%) | 119 (94.44%) | 7 (5.56%) | 0.0717 |
|  |  | 45-65 | 2366 (69.53%) | 2143 (91.74%) | 193 (8.26%) |  |
|  |  | >65 | 911 (26.77%) | 804 (89.63%) | 93 (10.37%) |  |
| Gender | 0 | Female | 1691 (49.69%) | 1532 (92.12%) | 131 (7.88%) | 0.0900 |
|  |  | Male | 1712 (50.31%) | 1534 (90.45%) | 162 (9.55%) |  |
| Race | 38 | Other | 697 (20.71%) | 622 (90.28%) | 67 (9.72%) | 0.2892 |
|  |  | White | 2668 (79.29%) | 2410 (91.57%) | 222 (8.43%) |  |
| Ethnicity | 94 | Hispanic or Latino | 403 (12.18%) | 356 (89.22%) | 43 (10.78%) | 0.1469 |
|  |  | Not Hispanic or Latino | 2906 (87.82%) | 2625 (91.50%) | 244 (8.50%) |  |
| Preferred Language | 4 | English | 3196 (94.03%) | 2885 (91.41%) | 271 (8.59%) | 0.2463 |
|  |  | Non-English | 203 (5.97%) | 177 (88.94%) | 22 (11.06%) |  |
| Insurance | 0 | Insured | 3270 (96.09%) | 2964 (91.74%) | 267 (8.26%) | **<.0001** |
|  |  | Underinsured | 133 (3.91%) | 102 (79.69%) | 26 (20.31%) |  |
| Colonoscopy Period | 0 | Pre-pandemic | 222 (6.52%) | 200 (91.32%) | 19 (8.68%) | 0.8348 |
|  |  | Pandemic | 150 (4.41%) | 138 (93.24%) | 10 (6.76%) |  |
|  |  | Post-pandemic | 235 (6.91%) | 214 (92.64%) | 17 (7.36%) |  |
|  |  | PN phase 1 | 1122 (32.97%) | 1014 (91.11%) | 99 (8.89%) |  |
|  |  | PN phase 2 | 1674 (49.19%) | 1500 (91.02%) | 148 (8.98%) |  |
| CRC Diagnosis | 0 | No | 3395 (99.76%) | 3059 (91.29%) | 292 (8.71%) | 1.0000 |
|  |  | Yes | 8 (0.24%) | 7 (87.50%) | 1 (12.50%) |  |
| *: For continuous variables, p-values were based on Wilcoxon rank sum test; for categorical variables, p-values were based on Chi-squared test with exact p-value from Monte Carlo simulation. Note: Median+/-IQR were reported for continuous variables; row percentages were reported for categorical variables. | | | | | | |

**Appendix 4. Comparison of the odds ratios for inadequate bowel preparation for screening/surveillance colonoscopy between underinsured and insured across the time periods after adjusting for age as a categorical variable, gender, and race, ethnicity, and preferred language.**

| **Level** | **Estimated ratios between underinsured vs insured (95% CI)** | **P-value** |
| --- | --- | --- |
| Pre-pandemic vs Pandemic | 4.78 (0.40, 57.04) | 0.2158 |
| Pre-pandemic vs Post-pandemic | 0.37 (0.07, 1.91) | 0.2324 |
| Pre-pandemic vs PN phase 1 | 3.97 (0.69, 22.85) | 0.1226 |
| Pre-pandemic vs PN phase 2 | 1.30 (0.30, 5.66) | 0.7290 |
| Pandemic vs Post-pandemic | **0.08 (0.01, 0.84)** | **0.0356** |
| Pandemic vs PN phase 1 | 0.83 (0.07, 9.78) | 0.8823 |
| Pandemic vs PN phase 2 | 0.27 (0.03, 2.65) | 0.2617 |
| Post-pandemic vs PN phase 1 | **10.85 (2.15, 54.89)** | **0.0039** |
| Post-pandemic vs PN phase 2 | 3.55 (0.94, 13.33) | 0.0610 |
| PN phase 1 vs PN phase 2 | 0.33 (0.08, 1.38) | 0.1287 |

**Appendix 5: Estimated coefficients for main effects from a multiple linear regression model analyzing the wait time (days) among screening/surveillance colonoscopy patients.**

| **Effect** | **Level** | **Estimated coefficients (95% CI)** | **P-value*** |
| --- | --- | --- | --- |
| Age (categorical) | <45 | -2.92 (-11.57, 5.73) | 0.0017 |
|  | 45-65 | -6.43 (-9.99, -2.87) |  |
|  | >65 | - |  |
| Gender | Male | 2.97 (-0.16, 6.11) | 0.0633 |
|  | Female | - |  |
| Race | White | 3.32 (-1.01, 7.65) | 0.1332 |
|  | Other | - |  |
| Ethnicity | Hispanic or Latino | 0.10 (-5.56, 5.76) | 0.9727 |
|  | Non-Hispanic or Latino | - |  |
| Preferred Language | English | 0.70 (-7.28, 8.68) | 0.8638 |
|  | Non-English | - |  |
| *: P-values were based on type 3 tests from a multiple linear regression model. Note: Other explanatory variables include insurance and colonoscopy period and the two-way interaction term. The corresponding estimated differences between underinsured and insured patients within each time period and across time periods are shown in Table 3 and Table 4, respectively. | | | |

**Appendix 6:** **Explanatory variables and their corresponding confidence intervals from a multivariable logistic regression model analyzing in having inadequate prep quality among screening/surveillance colonoscopy patients.**

| **Effect** | **Level** | **OR (95% CI)** | **P-value*** |
| --- | --- | --- | --- |
| Age (categorical) | 45-65 vs <45 | 1.46 (0.67, 3.18) | 0.0585 |
|  | >65 vs <45 | 1.93 (0.87, 4.29) |  |
| Gender | Male vs Female | 1.25 (0.97, 1.60) | 0.0790 |
| Race | Other vs White | 1.03 (0.73, 1.44) | 0.8791 |
| Ethnicity | Hispanic or Latino vs Not Hispanic or Latino | 1.18 (0.78, 1.81) | 0.4330 |
| Preferred Language | English vs Non-English | 1.30 (0.71, 2.38) | 0.3872 |
| Insurance*Colonoscopy Period | Underinsured vs Insured * 2019 | 4.56 (1.28, 16.24) | 0.0380 |
|  | Underinsured vs Insured * 2020 | 0.95 (0.11, 8.16) |  |
|  | Underinsured vs Insured * 2021 | 12.47 (4.08, 38.12) |  |
|  | Underinsured vs Insured * May 2022 - Oct 2022 | 1.15 (0.33, 4.04) |  |
|  | Underinsured vs Insured * Nov 2022 - May 2023 | 3.52 (1.56, 7.93) |  |
|  | 2019 vs Nov 2022 - May 2023 * Insured | 0.84 (0.48, 1.46) |  |
|  | 2020 vs Nov 2022 - May 2023 * Insured | 0.78 (0.39, 1.58) |  |
|  | 2021 vs Nov 2022 - May 2023 * Insured | 0.45 (0.22, 0.93) |  |
|  | May 2022 - Oct 2022 vs Nov 2022 - May 2023 * Insured | 1.03 (0.78, 1.35) |  |
|  | 2019 vs Nov 2022 - May 2023 * Underinsured | 1.08 (0.28, 4.24) |  |
|  | 2020 vs Nov 2022 - May 2023 * Underinsured | 0.21 (0.02, 1.85) |  |
|  | 2021 vs Nov 2022 - May 2023 * Underinsured | 1.60 (0.53, 4.82) |  |
|  | May 2022 - Oct 2022 vs Nov 2022 - May 2023 * Underinsured | 0.34 (0.08, 1.38) |  |
| * P-values were based on joint test from a multivariable logistic regression model. | | | |

**Appendix 7: Estimated coefficients for main effects from a multiple linear regression model analyzing the wait time (days) among diagnostic colonoscopy patients.**

| **Effect** | **Level** | **Estimated coefficients (95% CI)** | **P-value*** |
| --- | --- | --- | --- |
| Age (as a categorical variable) | <45 | -3.98 (-8.67, 0.72) | 0.0250 |
|  | 45-65 | 1.83 (-2.42, 6.08) |  |
|  | >65 | - |  |
| Gender | Male | -2.75 (-6.28, 0.79) | 0.1277 |
|  | Female | - |  |
| Race | White | -2.45 (-7.15, 2.24) | 0.3056 |
|  | Other | - |  |
| Ethnicity | Hispanic or Latino | 1.86 (-4.03, 7.74) | 0.5362 |
|  | Non-Hispanic or Latino | - |  |
| Preferred Language | English | 0.74 (-6.71, 8.19) | 0.8458 |
|  | Non-English | - |  |
| CRC Diagnosis | Yes | -21.13 (-35.60, -6.66) | 0.0042 |
|  | No | - |  |
| *: P-values were based on type 3 tests from a multiple linear regression model. Note: Other explanatory variables include insurance and colonoscopy period and the two-way interaction term. The corresponding estimated differences between underinsured and insured patients within each time period and across time periods are shown in Table 3 and Table 4, respectively. | | | |

**Appendix 8: Explanatory variables and their corresponding confidence intervals from a multivariable logistic regression model analyzing in having colonoscopy wait time>=60 days among diagnostic colonoscopy patients.**

| **Effect** | **Level** | **OR (95% CI)** | **P-value*** |
| --- | --- | --- | --- |
| Age (as a categorical variable) | 45-65 vs <45 | 1.28 (1.00, 1.65) | 0.0847 |
|  | >65 vs <45 | 1.33 (1.00, 1.76) |  |
| Gender | Female vs Male | 1.23 (0.99, 1.51) | 0.0579 |
| Race | Other vs White | 1.08 (0.82, 1.42) | 0.5746 |
| Ethnicity | Hispanic or Latino vs Not Hispanic or Latino | 1.18 (0.84, 1.66) | 0.3343 |
| Preferred Language | Non-English vs English | 1.07 (0.70, 1.64) | 0.7453 |
| Insurance*Colonoscopy Period | Underinsured vs Insured * 2019 | 1.65 (0.84, 3.24) | 0.0796 |
|  | Underinsured vs Insured * 2020 | 4.17 (1.81, 9.61) |  |
|  | Underinsured vs Insured * 2021 | 5.94 (2.88, 12.24) |  |
|  | Underinsured vs Insured * May 2022 - Oct 2022 | 2.21 (1.00, 4.88) |  |
|  | Underinsured vs Insured * Nov 2022 - May 2023 | 3.15 (1.46, 6.80) |  |
|  | 2019 vs Nov 2022 - May 2023 * Insured | 1.53 (1.08, 2.18) |  |
|  | 2020 vs Nov 2022 - May 2023 * Insured | 0.52 (0.31, 0.85) |  |
|  | 2021 vs Nov 2022 - May 2023 * Insured | 1.24 (0.85, 1.82) |  |
|  | May 2022 - Oct 2022 vs Nov 2022 - May 2023 * Insured | 1.10 (0.85, 1.43) |  |
|  | 2019 vs Nov 2022 - May 2023 * Underinsured | 0.80 (0.32, 2.01) |  |
|  | 2020 vs Nov 2022 - May 2023 * Underinsured | 0.68 (0.26, 1.78) |  |
|  | 2021 vs Nov 2022 - May 2023 * Underinsured | 2.35 (0.93, 5.93) |  |
|  | May 2022 - Oct 2022 vs Nov 2022 - May 2023 * Underinsured | 0.78 (0.28, 2.12) |  |
| CRC Diagnosis | No vs Yes | 3.61 (1.07, 12.20) | 0.0388 |
| * P-values were based on joint test from a multivariable logistic regression model. | | | |

**Appendix 9: Explanatory variables and their corresponding confidence intervals from a multivariable logistic regression model analyzing in having inadequate prep quality among diagnostic colonoscopy patients.**

| **Effect** | **Level** | **OR (95% CI)** | **P-value*** |
| --- | --- | --- | --- |
| Age (categorical) | 45-65 vs <45 | 1.14 (0.81, 1.60) | 0.5460 |
|  | >65 vs <45 | 1.23 (0.85, 1.79) |  |
| Gender | Male vs Female | 1.50 (1.14, 1.98) | 0.0040 |
| Race | Other vs White | 1.31 (0.92, 1.88) | 0.1335 |
| Ethnicity | Hispanic or Latino vs Not Hispanic or Latino | 1.18 (0.76, 1.84) | 0.4664 |
| Preferred Language | English vs Non-English | 1.20 (0.68, 2.11) | 0.5319 |
| Insurance*Colonoscopy Period | Underinsured vs Insured * 2019 | 1.34 (0.55, 3.24) | 0.1876 |
|  | Underinsured vs Insured * 2020 | 3.50 (1.28, 9.59) |  |
|  | Underinsured vs Insured * 2021 | 1.25 (0.49, 3.23) |  |
|  | Underinsured vs Insured * May 2022 - Oct 2022 | 0.79 (0.22, 2.85) |  |
|  | Underinsured vs Insured * Nov 2022 - May 2023 | 0.67 (0.22, 2.07) |  |
|  | 2019 vs Nov 2022 - May 2023 * Insured | 0.92 (0.57, 1.48) |  |
|  | 2020 vs Nov 2022 - May 2023 * Insured | 0.56 (0.29, 1.05) |  |
|  | 2021 vs Nov 2022 - May 2023 * Insured | 0.68 (0.39, 1.17) |  |
|  | May 2022 - Oct 2022 vs Nov 2022 - May 2023 * Insured | 0.68 (0.48, 0.96) |  |
|  | 2019 vs Nov 2022 - May 2023 * Underinsured | 1.84 (0.51, 6.67) |  |
|  | 2020 vs Nov 2022 - May 2023 * Underinsured | 2.90 (0.80, 10.57) |  |
|  | 2021 vs Nov 2022 - May 2023 * Underinsured | 1.27 (0.35, 4.63) |  |
|  | May 2022 - Oct 2022 vs Nov 2022 - May 2023 * Underinsured | 0.80 (0.16, 3.94) |  |
| CRC Diagnosis | Yes vs No | 1.28 (0.47, 3.49) | 0.6291 |
| * P-values were based on joint test from a multivariable logistic regression model. | | | |
